# The precision ceiling: information-theoretic accuracy bounds for responsible AI health screening

**DOI:** 10.3389/fdgth.2026.1956262

**Published:** 2026-09-21

**Authors:** Kai Ding, Zhi Li, Xiang Zou, Jingdong Jia, Chenlin Li, Zijing Jiang

**Affiliations:** 1 School of Physical Education, Xi’an University, Xi’an, Shaanxi Province, China; 2 Shaanxi Provincial Joint Laboratory of Artificial Intelligence, Xi’an University, Xi’an, Shaanxi Province, China; 3 School of Physical Education, Shaanxi Normal University, Xi’an, Shaanxi Province, China

**Keywords:** accuracy bounds, ceiling-aware design, data processing inequality, digital health ethics, health screening, precision ceiling, responsible artificial intelligence, sensing modality

## Abstract

Large language models pass licensing examinations and foundation models match human diagnostic accuracy, yet the sensing modalities that supply these algorithms with data obey unchanged physical limits. This article introduces the *precision ceiling*: the maximum accuracy attainable by any model operating on a given sensing modality, bounded by the information-theoretic capacity of the measurement channel rather than by algorithmic capacity. Grounded in the data processing inequality, the ceiling is an *a priori* constraint whose clinical magnitude must be empirically characterized per modality. We develop a three-layer nested constraint taxonomy (Physical Law → Physiological Variability → Modality Gap), demonstrate the phenomenon across four optical and photoelectric sensing applications (postural screening, wearable photoplethysmography, fundus imaging, and dermatological AI), and propose two conceptual design contributions—the IICRC design principles (Identify, Internalize, Communicate, Recognize, Cascade) and the Precision Ceiling Audit, a qualitative pre-deployment characterization template—offered for future empirical validation. The framework does not replace existing responsible-AI initiatives; it identifies where humility and safety are physically inevitable rather than aspirational, providing a principled basis for modality-specific accuracy declarations, intended-use restrictions, and escalation protocols aligned with emerging regulatory expectations.

## 1 Introduction

AI-enabled health screening devices are entering clinical practice at accelerating volume. Large language models now pass the United States Medical Licensing Examination (1); foundation models achieve human-level diagnostic accuracy across imaging modalities (2); and the pipeline of AI-enabled medical devices cleared by the US Food and Drug Administration (FDA) and notified under the European Union Medical Device Regulation (EU MDR) continues to grow (3). The World Health Organization’s global strategy on digital health 2020–2025 explicitly positions AI-enabled screening as a priority pathway for strengthening health systems (4). The field expects that more data, larger models, and better algorithms will eventually close the remaining accuracy gaps.

This expectation contains a category error. Algorithmic sophistication improves the extraction of information from a given signal; it cannot recover information that was never captured by the sensor. When a structured-light surface topography system attempts to infer three-dimensional (3D) spinal geometry from the back surface, or when a wrist-worn photoplethysmographic (PPG) sensor attempts to characterize beat-to-beat cardiac rhythm regularity, or when a dermoscopic image is tasked with revealing the depth of a subcutaneous lesion—in each case, the clinical parameter of interest lies on the far side of a physical interface that the sensing modality cannot fully cross. Under the data processing inequality, no improvement in model architecture, training data volume, or computational scale can, in principle, manufacture information that the measurement channel did not preserve.

Three active lines of work have independently approached this problem from different angles. The uncertainty quantification (UQ) community has developed methods for distinguishing aleatoric from epistemic uncertainty in medical AI (5, 6). The abstention and selective prediction community—building on foundational work in uncertainty-aware classification (7) and experiencing a burst of activity in 2025–2026 with frameworks such as DEGRE, KnowGuard, ClinDet-Bench, and HUQ-2 (8–11)—has advanced the capacity for models to refuse outputs when confidence is low. The responsible AI community has articulated ethical frameworks, from BODHI’s humility checkpoints (12) to FUTURE-AI’s governance principles (13) to Müller et al.’s “Seven Deadly Sins” of digital medicine (14).

All three lines address *how to respond* to accuracy limits; none addresses *where those limits originate*. Yet across all three, a shared structural phenomenon remains unnamed. Each community has diagnosed a symptom—statistical noise, overconfident outputs, ethical overreach—without identifying their common physical root: the information bottleneck at the sensor-tissue interface. Bartlett et al. (15) came closest when they demonstrated that invasive measurements could define an objective accuracy ceiling for non-invasive models (15), but their framework was limited to a single invasive/non-invasive dichotomy applied during model training. Zhu et al. (16) introduced “Performance Ceilings” in clinical EEG classification (16), but showed that these ceilings—rooted in label noise and inter-rater agreement (∼86%–90%)—could be surpassed by improved architectures, reaching 99% accuracy.

This article identifies a different kind of ceiling—one rooted not in statistics or deployment but in sensing physics. We introduce the **precision ceiling** (defined formally in Section 2.1) and develop its implications at three levels: **(1) conceptual structure**, through a three-layer nested constraint taxonomy; **(2) practical response**, through the IICRC framework of ceiling-aware design principles; and **(3) design translation**, through the Precision Ceiling Audit. The framework is intended to provide digital health researchers, developers, and regulators with a physically grounded vocabulary for identifying where AI screening systems must acknowledge their limits—and where responsible deployment requires architectural humility rather than algorithmic ambition.

## 2 The precision ceiling: definition and taxonomy

### 2.1 Definition and scope

The precision ceiling arises from a structural information asymmetry between the physical reality a sensor attempts to measure and the signal it actually captures. Consider postural screening: two different vertebral rotation configurations can produce nearly identical back-surface deformations, so no algorithm can determine which configuration is present from the surface alone. Formally, the sensing modality maps a physical phenomenon onto a signal space through a non-injective (many-to-one) transformation—whether by dimensional reduction, projection geometry, or physical attenuation—so that distinct physical states produce indistinguishable sensor outputs. This is a consequence of the data processing inequality (DPI): for any Markov chain X→Y→Z, the mutual information satisfies I(X;Z)≤I(X;Y), meaning no post-processing can increase the Shannon information about the original quantity beyond what the measurement channel preserves (17, 18). In the sensing context, physical reality → sensor signal → model output forms such a chain, so no architecture can recover, in the Shannon sense, what the channel did not transmit. Importantly, the DPI bounds information, not accuracy directly; a further step is needed to translate an information bound into a bound on estimation or classification error. Classical results supply this bridge: Fano’s inequality lower-bounds the probability of error of any estimator of X from Y in terms of the conditional entropy H(X∣Y) (17), and information–error relations connect I(X;Y) to the Bayes error rate (19). The precision ceiling therefore rests on two pillars: an information bound on the channel (the DPI) and the task-relevance assumption that the discarded information is the part required for the clinical decision. Dimensional reduction (N>M) is one sufficient condition for such information loss, but non-injectivity can arise even without dimensional reduction—whenever the sensor-to-target mapping is non-unique.

The DPI is a theorem about Shannon (syntactic) information—the statistical dependence between signal distributions. The precision ceiling’s clinical relevance depends on the additional, empirically testable assumption that the lost Shannon information carries clinically discriminative features. When this assumption holds—when the information the sensor fails to capture includes features necessary for the clinical decision—the DPI provides a principled bound. When it does not hold—when the lost information is clinically irrelevant—the Shannon bound has no operational consequence. The precision ceiling framework thus combines two components: **(a)** an *a priori*, pre-model conceptual derivation from the DPI that establishes *that* information loss occurs at non-injective sensor interfaces, and **(b)** an *a posteriori*, empirically grounded characterization of *which* clinical parameters are affected, *how severely*, and whether the ceiling falls below the clinical utility threshold.

A formal probabilistic model makes the construct precise. Let T denote the latent physiological state that is the clinical target (e.g., true axial vertebral rotation), X the physical quantity a sensor is designed to probe, Y the sensor observation, and Z the model output. Validation uses a reference standard L (e.g., CT-derived rotation), which is a noisy observation of T rather than an error-free ground truth. The generative structure is T→X→Y→Z, with L an additional noisy copy of T. The channel of clinical interest is the composite mapping T→Y, which decomposes into a projection/physiological stage T→X (how the target expresses itself at the accessible surface) and a transduction stage X→Y (how the sensor records that surface). Because T→Y→Z is a Markov chain, the DPI gives I(T;Z)≤I(T;Y)≤I(T;X) (17): no model can increase the information about the clinical target T present in the observation Y. When the composite channel T→Y is non-injective—whether through the projection T→X or the transduction X→Y—H(T∣Y)>0, and information–error bounds imply a positive floor on the error of any estimator of T from Y (17, 19). These two stages align with the taxonomy of Section 2.3: the transduction X→Y carries the Physical Law constraints of Layer 1, whereas the projection T→X, modulated by the Physiological Variability of Layer 2, is where the Layer 3 Modality Gap originates.

We emphasize the relationship between the precision ceiling and the Bayes error rate. The Bayes error rate conditional on the observed features—the minimum error achievable by any classifier of T from Y—already reflects the sensing channel, because the feature distribution P(Y) is itself produced by that channel: whatever information that composite channel discards reappears as irreducible residual uncertainty in P(T∣Y). The precision ceiling therefore does not supply a bound that is numerically tighter than, or otherwise distinct from, the Bayes error rate evaluated on the channel-conditional distribution. Its contribution is epistemic rather than purely mathematical, in two respects. **First (causal attribution)**, it decomposes the Bayes error into the component imposed by the measurement channel—the part that persists as data volume, model capacity, and compute grow without bound—vs. components attributable to finite sampling, label noise, or algorithmic capacity. **Second (prospective predictability)**, because the channel component is a function of the sensor’s physical specification (optical penetration depth, projection geometry, diffraction limit), it can be predicted before any training data are collected, whereas the Bayes error rate itself can only be estimated a posteriori from an observed distribution. This is the precise meaning of “pre-statistical”: the ceiling is derived from the physics of the channel and the task relevance of the discarded information, not estimated from a data distribution.

In clinical terms, the precision ceiling describes the irreducible gap between the surrogate measurement a sensor captures—3D back surface topography, dermoscopic photographs, PPG waveforms—and the clinical parameter of interest: 3D skeletal geometry, lesion depth, cardiac rhythm regularity. As the model above makes explicit, the precision ceiling is not a quantity separate from the Bayes error rate but the physically attributable, prospectively predictable component of it—the part of the irreducible error that is imposed by the sensing channel before any distribution is sampled.

The term “accuracy” here requires qualification. When the clinical target is structurally inaccessible through the sensing channel—as with vertebral rotation via surface topography, lesion depth via dermoscopy, or cardiac rhythm via PPG—“accuracy” means agreement between the sensor-derived estimate and a reference-standard measurement (e.g., CT for vertebral rotation, histopathology for lesion depth, electrocardiogram for rhythm), not correspondence to a mind-independent true value. This distinction aligns with the metrological concept of traceability (20) and with construct-validity frameworks in clinical measurement (21, 22), which we address further in Section 2.4.

“The precision ceiling is the maximum accuracy attainable by any model operating on a given sensing modality—in the limit of infinite data and compute, set not by algorithmic capacity but by the information-theoretic limits of the measurement channel itself.”

Importantly, this claim is bounded. This article provides a conceptual framework and a qualitative discrimination tool for identifying structural accuracy bounds—not a method for computing numerical ceiling values. A precision ceiling constitutes a practical constraint, however, only when it falls below the **clinical utility threshold** for the target parameter: a ceiling of ±3 bpm for heart rate monitoring is clinically irrelevant if the clinical requirement is ±5 bpm. The concept becomes consequential precisely when the physically attainable accuracy is insufficient for the clinical task.

### 2.2 Distinction from related concepts

#### 2.2.1 Precision ceiling vs. aleatoric uncertainty

Aleatoric uncertainty is the Bayes error rate conditional on the observed features—the irreducible error of the optimal classifier given the data distribution, estimated a posteriori. The precision ceiling is the component of that irreducible error imposed by the sensing channel, identified by physical analysis of the channel and predictable *a priori*. The two are not mutually exclusive: the precision ceiling is a causally attributed, prospectively predictable part of the aleatoric uncertainty, whereas aleatoric uncertainty as usually estimated is an undifferentiated, a posteriori measure of all residual error. Aleatoric uncertainty asks: “Given these observed features, what is the best accuracy any classifier can achieve?” The precision ceiling asks: “Given this sensor, how much of that irreducible error is physically unavoidable and knowable before any data are collected?”

#### 2.2.2 Precision ceiling ≠ statistical performance ceilings

Bartlett et al. (15) demonstrated that invasive measurements can define an objective accuracy ceiling for non-invasive models (15). We generalize this insight along two axes: from a single invasive/non-invasive dichotomy to a framework spanning multiple sensing modalities; and from a training-phase diagnostic to a design-phase principle. Zhu et al. (16) introduced “Performance Ceilings” in clinical EEG classification—limits rooted in label noise (∼86%–90%) (16). Critically, these ceilings were *broken*: improved architectures achieved 99% accuracy, demonstrating that the original ceiling was a statistical artifact, not a physical constraint. The precision ceiling, by contrast, cannot be surpassed without modality change—only acknowledged and operationally addressed. We term the discrimination tool the **Ceiling Shift Test**: if a reported accuracy improvement can be attributed to better hardware, more data, or improved architecture, the original bound was a technical limitation. If the improvement requires changing the sensing modality, the original bound was a genuine precision ceiling.

#### 2.2.3 Precision ceiling ≠ deployment or architectural gaps

Ivchenko’s “8× Gap” (23) and Ingrisch’s “architectural gap” in *The Lancet Digital Health* (24) diagnose performance degradation between laboratory benchmarks and real-world deployment. These are deployment-layer phenomena, addressable through better system engineering. The precision ceiling operates at a deeper layer: the information content of the signal itself. A summary comparison is provided in Table 1.

**Table 1:** Distinction between the precision ceiling and related accuracy-bound concepts.

Dimension	Precision ceiling	Aleatoric uncertainty	Bartlett ceiling (15)	Zhu ceiling (16)	Deployment gap (23, 24)
Definition	Information-theoretic upper bound of the sensing channel	Irreducible noise within a feature distribution	Bound defined by invasive measurement	Bound defined by label noise	Lab-to-clinic performance loss
Origin	Sensing physics	Data distribution	Modality hierarchy	Annotation quality	Data drift / integration
Timing	Before data collection	After data collection	During training	During evaluation	After deployment
Breakable?	No (modality change required)	Yes (improved SNR)	N/A (training criterion)	Yes (86%→99%)	Yes (engineering)
Response	IICRC + Audit	Improved UQ	Early stopping	Better architecture	Better pipeline

### 2.3 A three-layer nested constraint hierarchy

The precision ceiling operates as a nested constraint hierarchy of three layers, progressing from fundamental physical origin to clinical manifestation (Figure 1):

**Figure 1:**
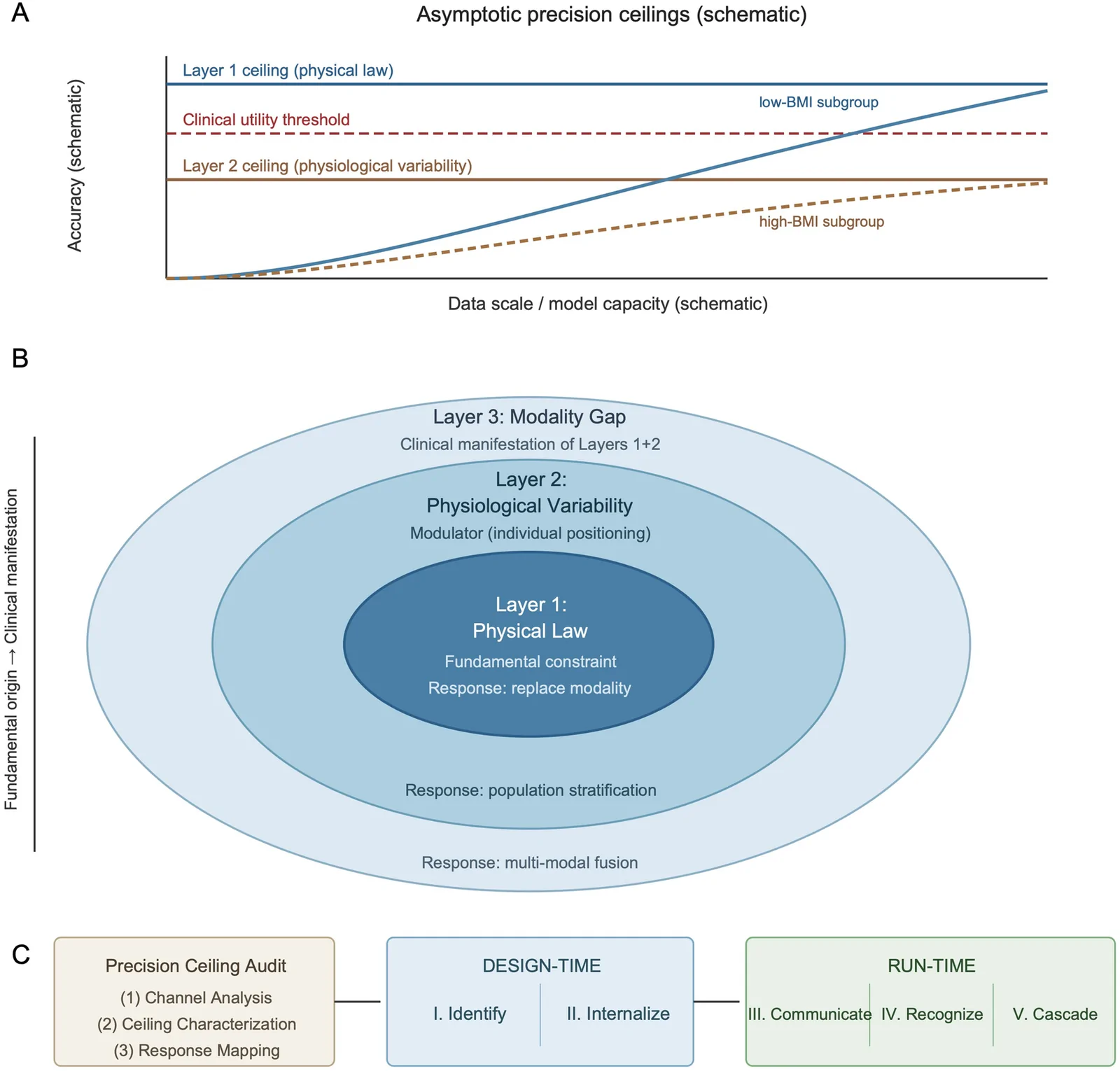
The precision ceiling framework. **(A)** Asymptotic precision ceilings (schematic): as data scale and model capacity grow, achievable accuracy approaches a horizontal asymptote—the Layer 1 ceiling (physical law) for a low-BMI subgroup, or a lower Layer 2 ceiling (physiological variability) for a high-BMI subgroup—relative to the clinical utility threshold (dashed). **(B)** Three-layer nested constraint hierarchy: Layer 1—Physical Law (innermost) → Layer 2—Physiological Variability (modulator) → Layer 3—Modality Gap (outermost; clinical manifestation). Each layer maps to a distinct design response. **(C)** IICRC framework organized across two operational layers: Design-Time (I. Identify → II. Internalize) and Run-Time (III. Communicate → IV. Recognize → V. Cascade), with the Precision Ceiling Audit as the pre-deployment entry point.

#### 2.3.1 Layer 1: Physical law—the fundamental constraint

Absolute physical bounds: optical attenuation and scattering in biological tissue—described by the radiative transfer equation and its diffusion approximation, of which the (modified) Beer-Lambert law is a limiting case—the diffraction limit of optical imaging, and quantum noise in radiation-based modalities. *Diagnostic implication:* replace or supplement the modality.

#### 2.3.2 Layer 2: Physiological variability—the modulator

Physical laws manifest differently across individuals. Soft tissue thickness amplifies the surface-to-skeleton gap; lens opacity amplifies photon scattering; melanin concentration amplifies signal attenuation. *Diagnostic implication:* population stratification—declaring accuracy bounds within specified BMI or skin phototype ranges.

#### 2.3.3 Layer 3: Modality gap—the clinical manifestation

The downstream clinical expression of Layer 1 as modulated by Layer 2. *Diagnostic implication:* multi-modal fusion may partially recover lost information—though complete elimination is not achievable within a fixed sensor configuration.

We note a scope boundary: signal-processing degradation (quantization noise, compression artifacts) represents engineering choices alterable without modality change and thus does not constitute a precision ceiling under the Ceiling Shift Test.

##### 2.3.3.1 Falsifiability

The falsification condition is: a model operating on a given sensing modality surpasses the accuracy bound predicted from the channel’s information capacity—without changing the sensing modality or incorporating additional information sources. If such a demonstration were produced, the ceiling classification for that application would be falsified.

### 2.4 Measurement foundations: accuracy, construct validity, and the precision ceiling

The precision ceiling framework rests on a correspondence model of measurement appropriate for physically defined quantities traceable to reference standards (20). However, clinical measurement also includes constructs whose definition is partly constituted by the measurement procedures used to assess them (21, 22). For example, “functional impairment” is validated not against a single gold standard but through a network of convergent assessments; the precision ceiling does not apply to such constructs directly. The ceiling and construct validity operate at complementary levels: the ceiling describes the maximum fidelity of the sensor-to-signal mapping; construct validity addresses whether the signal adequately represents the clinical construct. The ceiling applies to the physical signal, not to the construct validity of the clinical target.

Following the VIM (20), we fix the meanings of four terms used throughout. *Accuracy* is the closeness of agreement between a measured quantity value and a reference quantity value; here the reference is the reference-standard measurement (e.g., CT, ECG, histopathology) specified in Section 2.1. *Trueness* is the closeness of agreement between the average of replicate measurements and the reference value (the complement of systematic bias). *Precision*, in the VIM, is the closeness of agreement among replicate measurements—a dispersion concept (repeatability/reproducibility), not an accuracy concept. The name *precision ceiling* therefore carries an explicit caveat: it uses “precision” in its colloquial sense of “the attainable fidelity of knowledge”—what the VIM would classify as a bound on accuracy, defined against a reference standard—rather than as a dispersion. Finally, *sensitivity* is used throughout in its diagnostic sense (true-positive rate), distinct from metrological sensitivity (the change in instrument output per unit change in the measured quantity).

## 3 Anchor case: postural screening and the surface-skeletal gap

### 3.1 A textbook ceiling

That surface topography cannot perfectly capture internal skeletal geometry is well known in orthopedics. The SOSORT consensus guidelines characterize surface topography as a supplementary tool that can reduce—but should not replace—radiographic examination (25). Three independent systematic reviews (two with meta-analytic pooling), collectively spanning over five thousand patients, converge on a consistent evidence pattern. Bertsch et al. (26) (56 studies, 4,774 patients) reported pooled correlations exceeding r>0.8 for coronal plane parameters (26). Li et al. (27) confirmed “sufficient” criterion validity across 23 studies (27). Su et al. (28) found good-to-excellent reliability for most surface-topography measurements (28).

### 3.2 Why the gap cannot be closed

The residual error displays three signatures inconsistent with a purely statistical noise floor:

#### 3.2.1 Equivocation (overlapping surface morphology)

Distinct vertebral configurations—different combinations of axial rotation, lateral translation, and soft-tissue distribution—produce overlapping, near-identical back-surface morphologies, so the surface does not determine the underlying geometry uniquely. This many-to-one mapping is precisely the non-injectivity that the DPI identifies as the source of an irreducible bound: when multiple skeletal states map to the same surface observation, no post-processing can recover which state produced it (17). A reported tendency for surface topography to underestimate the Cobb angle relative to radiography, with limited evidence quality for severe curves and higher-BMI patients (26, 27), should not be read as independent evidence for a ceiling: a purely systematic directional bias is, in principle, post-hoc calibratable, and would not by itself distinguish a physical ceiling from a correctable technical limitation. That tendency may partly reflect the equivocation of the mapping, but what cannot be calibrated away is the equivocation itself.

#### 3.2.2 Dimension selectivity

Vertebral rotation is rated as having insufficient validity against CT/MRI across all three reviews (26–28), while coronal parameters achieve sufficient validity—the pattern predicted when a measurement channel is selectively non-injective.

#### 3.2.3 BMI dependence

All three reviews note evidence quality degrades at higher BMI (26–28)—the Physiological Variability layer modulating the Physical Law constraint.

A deep learning study by Minotti et al. (29) demonstrated that CNNs improve Cobb angle prediction from surface topography (29)—but the structured error patterns persist. The model approaches the ceiling; it does not break through it.

### 3.3 Clinical implications

In ceiling-aware screening systems, the AI output is deliberately constrained: it reports a risk grade rather than a Cobb angle estimate. The SOSORT guidelines independently support this triage-oriented role (25), providing convergent clinical validation for the Recognize-and-Cascade protocol.

## 4 Generalization across optical and photoelectric sensing

### 4.1 Wearable photoplethysmography

Wearable PPG sensors face a precision ceiling rooted directly in Physical Law. Light transport in tissue is governed by the radiative transfer equation and its diffusion approximation (30): green light (≈530 nm) penetrates approximately 1–2 mm, while red and near-infrared wavelengths reach 3–4 mm in scattering tissue (31); Monte Carlo simulations confirm these depth limits (32). The relevant precision ceiling for PPG AF detection concerns: (a) the fidelity with which the mechanical pulse wave preserves beat-to-beat interval information; (b) the signal-to-noise ratio floor imposed by motion artifact; and (c) pulse deficits creating a hard physiological ceiling for any optical method. Antiperovitch et al. (33) demonstrated that deep learning improves PPG-based AF detection by classifying substantially more of the recorded signal than heuristic processing, while remaining below the accuracy of ECG reference recordings (33). A 2025 meta-analysis of 15 studies reported pooled PPG smartwatch sensitivity of 97.4% (95% CI 96.5%–98.3%) for AF detection, statistically indistinguishable from single-lead ECG smart chest patches (34)—both wearable channels remain below standard 12-lead ECG diagnostics, a gap no architecture within the PPG modality can close.

### 4.2 Fundus imaging

When ocular media are transparent, AI-based DR screening achieves sensitivities exceeding 90% (35). A distinct phenomenon arises when media become opaque: 10%–30% of fundus photographs are ungradable (36)—complete signal failure rather than a precision ceiling. For the gradable subset, however, a candidate ceiling persists: two-dimensional fundus photographs compress a three-dimensional retinal vasculature onto a plane, so depth information (e.g., early neovascular elevation) is structurally attenuated. The fundus case thus illustrates the diagnostic value of the Ceiling Shift Test: signal failure (ungradable images) is technically resolvable and lies outside the framework; depth compression in gradable images is a candidate precision ceiling that optical coherence tomography—a modality change—can address but fundus photography cannot.

### 4.3 Dermatological AI

Standard dermoscopy captures visible-light reflectance with an effective penetration depth of approximately 1–2 mm (37). Performance degrades on darker skin phototypes due to training data imbalance (38). Data bias is addressable through inclusive data collection; the precision ceiling, rooted in optical penetration depth, is not.

### 4.4 Pattern, boundary, and evidence hierarchy

The four cases share a common information architecture (Figure 2): in each, the sensor records a surrogate surface while the clinical parameter lies beyond a physical interface. Boundary cases—multispectral imaging (shifts the ceiling upward without eliminating it) and computational imaging (operates within or augments the measurement channel)—demonstrate that the precision ceiling is a discriminating concept, not a tautology. This is not an argument against algorithmic progress: advances such as graph-guided weakly supervised learning for tumor-region discovery (39) and deeper analyses of network internal representations (40) improve how efficiently the available signal is extracted and understood, but they operate on the channel’s output and remain bounded by it. Wound photography (Figure 2, bottom row) illustrates a parameter-level boundary case: wound surface area is directly accessible to the camera and passes the Ceiling Shift Test, whereas wound depth and undermining lie behind the tissue interface and are therefore ceiling-bound for two-dimensional photography. The same application is thus simultaneously non-ceiling for one parameter and ceiling-bound for another.

**Figure 2:**
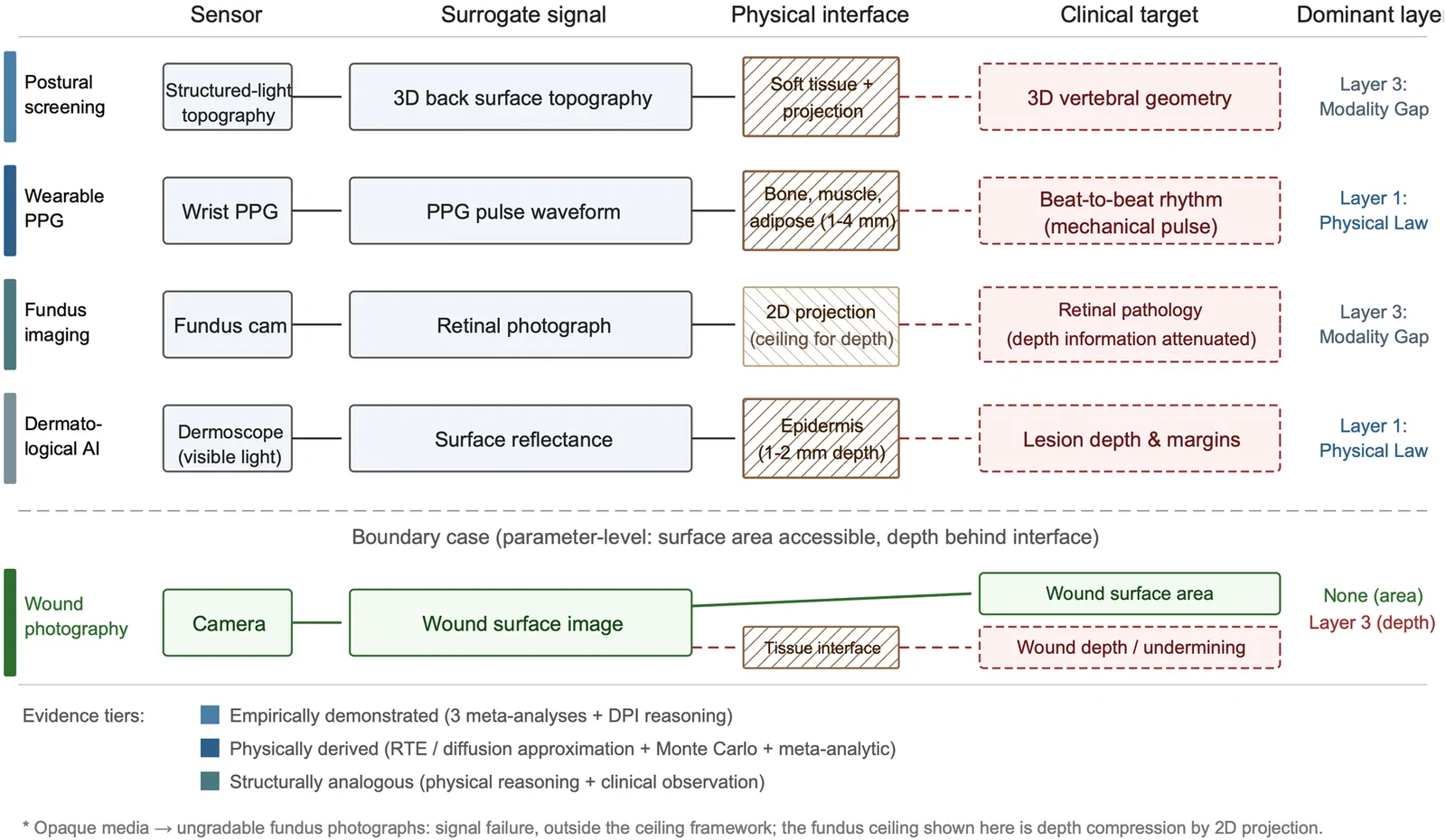
Cross-application information architecture. Each row depicts one sensing application: Sensor → Surrogate signal → Physical interface (hatched barrier) → Clinical target. Dashed arrows indicate irretrievable information loss; the hatched region marks the precision ceiling locus. The bottom row (wound photography) illustrates a parameter-level boundary case: wound surface area is directly accessible (no interface, green), whereas wound depth and undermining lie behind the tissue interface (hatched) and are ceiling-bound (red). Evidence hierarchy: postural screening—empirically demonstrated; PPG—physically derived; fundus imaging and dermatological AI—structurally analogous.

We acknowledge that the evidence is asymmetric: postural screening is **empirically demonstrated**; PPG is **physically derived** with convergent meta-analytic support; fundus imaging and dermatological AI are **structurally analogous**.

## 5 Implications: ceiling-aware system design

### 5.1 The IICRC framework

Existing responsible-AI frameworks have established that systems should be humble (12), trustworthy (13), and free of false precision (14)—but none specifies *where* these virtues become physical necessity. The IICRC framework supplies this missing link by translating the precision ceiling’s physical analysis into proposed design principles. We emphasize that IICRC is a conceptual framework: its principles are grounded in the physical analysis above, but their operational effectiveness requires empirical validation through prospective implementation studies.

#### 5.1.1 Design-time layer

##### 5.1.1.1 Identify

Distinguish physical precision ceilings from temporary technical limitations using the Ceiling Shift Test, applied prospectively from first-principles channel analysis.

##### 5.1.1.2 Internalize

Encode the ceiling into the system architecture at three levels: (a) data pipeline input-quality thresholds; (b) model output-head precision bounds; (c) deployment intended-use population restrictions.

#### 5.1.2 Run-time layer

##### 5.1.2.1 Communicate

Convey uncertainty along two dimensions: degree (distance from ceiling) and nature (sensor noise vs. out-of-distribution input vs. irreducible information gap).

##### 5.1.2.2 Recognize

When the precision ceiling falls below the clinical utility threshold, constrain the system’s output. Unlike confidence-based abstention (8–11), Recognize is triggered by the structural information deficit itself.

##### 5.1.2.3 Cascade

Transfer judgment, with a documented ceiling analysis, to a capable human or alternative system. For postural screening: risk grade → radiographic referral. For PPG: irregular-pulse probability → confirmatory ECG. For dermatological AI: lesion-risk category → biopsy recommendation.

### 5.2 The precision ceiling audit

We propose the **Precision Ceiling Audit**: a qualitative three-step characterization template intended to structure ceiling analysis before model development. It is offered as a conceptual design aid—a structured reasoning scaffold—rather than as a validated operational workflow.

**Step 1: Channel analysis.** Map the measurement channel; at each transformation, identify whether information loss is recoverable or structural.

**Step 2: Ceiling characterization.** Using sensor specifications, physical first principles, and published validation studies, characterize the ceiling qualitatively and, where evidence permits, semi-quantitatively.

**Step 3: Response mapping.** Compare the characterized ceiling to the clinical utility threshold. If below, define the Recognize-and-Cascade protocol.

*Worked prototype (postural screening).* Coronal Cobb angle from surface topography illustrates a semi-quantitative ceiling characterization. First principles identify the surface-to-skeleton mapping as non-injective for axial rotation but approximately injective for coronal curvature (Section 3.2); published validation studies then anchor the ceiling empirically, with pooled surface-to-radiographic agreement of r>0.8 for coronal parameters (26, 27), alongside reported residual measurement error on the order of $\pm 5^{\circ }$–$7^{\circ }$ relative to radiography. Setting this against a clinically meaningful progression threshold of approximately $\pm 5^{\circ }$ (41) places the coronal ceiling at—not comfortably above—the clinical utility threshold, warranting risk-graded rather than numerical output, whereas the insufficient validity of axial rotation (26–28) places that ceiling below the screening threshold, indicating Recognize-and-Cascade. This prototype is deliberately semi-quantitative: it combines a physical argument (which mappings are non-injective) with published validation ranges and a clinical threshold, rather than computing a channel-capacity bound. It is offered as the template for the application-specific ceiling characterization on which any judgment that a model is near-optimal would have to rest.

The Audit reorients the AI development workflow: not “how accurate is our model?” but “what accuracy does the signal permit?” This logic is consistent with FDA intended-use declarations and EU MDR clinical performance evaluations (3).

Table 2 illustrates, for the anchor case, how a ceiling characterization translates into concrete design changes across the dimensions that define a system’s clinical role.

**Table 2:** Worked case: how ceiling characterization changes the design of a postural screening system.

Design element	Without ceiling awareness	With ceiling awareness (Recognize-and-Cascade)
Intended use	Cobb angle estimation for all patients	Risk grading within a validated BMI range; no numerical Cobb angle
Model output	Numerical Cobb angle estimate	Risk category (low/medium/high) with a documented precision bound
Referral threshold	None specified	Medium or high risk triggers radiographic referral
Decision pathway	Surface topography interpreted as diagnostic	Surface topography as triage; radiography for ceiling-bound parameters (e.g., axial rotation)
Population restriction	None	Documented BMI range; out-of-range patients cascaded

### 5.3 Cost-effectiveness and the cascade decision

Ceiling-aware design carries an inherent tension: correctly identifying a ceiling may systematically route patients toward higher-cost confirmatory pathways. A natural decision rule is to cascade when the expected harm of a ceiling-bound error exceeds the expected burden of referral (Equation 1):

$$P(\text{error}|\text{ceiling})\times C_{\text{error}}>P(\text{complication}|\text{cascade})\times C_{\text{complication}}+C_{\text{cascade}}$$ (1)

where C denotes application-specific costs (clinical, economic, or utility-weighted). Quantitative parameterization of this inequality—drawing on epidemiological base rates, cascade complication data, and health-economic valuations—is a necessary companion analysis for any deployment; we flag it here as a structural requirement of ceiling-aware design rather than attempting generic calibration.

## 6 Discussion

### 6.1 Summary and significance

This article has introduced the precision ceiling—a physically grounded accuracy bound arising from the information-theoretic limits of the sensing channel—and developed its implications for responsible AI health screening. A model that achieves 90% accuracy on a task with a 92% ceiling is near-optimal; a model that achieves 90% on a task with a 99% ceiling is underperforming. This example is illustrative rather than operational: because the framework does not yet supply a general computational method for ceiling values, distinguishing these two scenarios in practice currently requires the application-specific, semi-quantitative characterization of Section 5.2. The concept’s contribution is therefore not a ready-made threshold but a question—“what does the signal permit?”—that makes the distinction principled even where a numerical ceiling remains to be estimated.

The precision ceiling does not replace existing responsible-AI frameworks—it grounds them. BODHI says “be humble” (12); the ceiling shows where humility is physically compelled. FUTURE-AI says “be safe” (13); the ceiling shows that safety at the physical boundary demands a specific architectural response—output constriction and cascade—rather than a general exhortation. The practical shift is from aspirational principle to engineering specification: the locus of responsibility moves from the ethicist’s checklist to the sensor physicist’s channel analysis.

### 6.2 Strengths and limitations

The principal strength is unification: observations previously treated in isolation—statistical noise floors, overconfident outputs, ethical overreach—fall under a single physical explanation with a common diagnostic tool (the Ceiling Shift Test) and a common design architecture (IICRC + Audit).

The framework has five limitations. **First**, as noted in Section 2.1, it is conceptual and qualitative; it does not provide a computational method for deriving numerical ceiling values. **Second**, the evidence base is asymmetric across applications: the postural screening case is meta-analytically confirmed, PPG is physically derived with convergent meta-analytic support, while fundus imaging and dermatological AI remain structurally analogous, and their ceilings await systematic, meta-analytic quantification. **Third**, the framework is currently restricted to optical and photoelectric modalities. **Fourth**, the IICRC framework and Precision Ceiling Audit are proposed conceptual design principles rather than validated operational tools; their effectiveness remains to be demonstrated through prospective implementation studies. **Fifth**, the cost-effectiveness analysis remains qualitative.

### 6.3 Testability and falsifiability

As stated in Section 2.3, the framework is falsifiable: a model surpassing the predicted bound without modality change would overturn a claimed ceiling. Operationalizing this test requires specifying channel capacity for a given clinical parameter—non-trivial, but the logical standard is clear. Claimed precision ceilings should be treated as hypotheses to be tested, not as immutable verdicts.

### 6.4 Implications for digital health governance and regulation

The EU MDR (Regulation (EU) 2017/745) requires AI-enabled medical devices to declare intended use and demonstrate clinical performance within specified operating conditions; the FDA’s predetermined change control framework imposes analogous boundaries (3). The precision ceiling provides a physically principled basis for these declarations: the intended-use statement of a ceiling-bound device should explicitly acknowledge the sensing modality’s information limits, specify population subgroups where the ceiling degrades, and document escalation pathways. More broadly, the framework identifies the loci where responsible design is not ethical aspiration but physical inevitability, enabling regulators to concentrate governance resources where they matter most.

### 6.5 Future directions

We identify five research priorities: (1) quantitative ceiling estimation via information-theoretic channel capacity methods, building on the probabilistic model of Section 2.1 to compute channel-conditional Bayes error bounds for defined clinical parameters, together with systematic ceiling quantification for the structurally analogous cases (fundus imaging, dermatological AI); (2) empirical validation of IICRC through prospective implementation studies; (3) extension to non-optical modalities (ultrasound, MRI, electrochemical sensing); (4) integration with regulatory science (EU MDR, FDA pre-market submissions); (5) health-economic modeling to parameterize the cascade decision principle.

### 6.6 Concluding remarks

Two cautions accompany the framework. First, it must not become a justification for underinvestment in algorithmic improvement where genuine headroom exists, nor for neglecting sensor hardware development that could shift ceilings upward. The precision ceiling identifies where algorithmic improvement yields diminishing returns; it does not foreclose improvement in the sensing modality itself. Second, claiming a ceiling is a falsifiable empirical commitment, not a permanent exemption from further validation.

The operational question for digital health shifts accordingly: before asking how accurate a model can become, ask what accuracy the signal permits.

## Data Availability

The original contributions presented in the study are included in the article/Supplementary Material, further inquiries can be directed to the corresponding authors.
